# Effectiveness of Conventional Swallowing Therapy in Acute Stroke Patients with Dysphagia

**DOI:** 10.1155/2020/2907293

**Published:** 2020-10-05

**Authors:** Hathaya Jongprasitkul, Wasuwat Kitisomprayoonkul

**Affiliations:** Department of Rehabilitation Medicine, Faculty of Medicine, Chulalongkorn University, Bangkok, Thailand

## Abstract

**Background:**

Dysphagia is a common problem in acute stroke patient. Aspiration pneumonia increases in this group. Swallowing therapy is immediately conducted in a stable stroke patient. An effectiveness of our program has not been determined.

**Objective:**

To determine an effectiveness of conventional swallowing therapy in acute stroke patients with dysphagia.

**Methods:**

We retrospectively reviewed data from medical records of acute stroke patients with dysphagia who participated a swallowing therapy from January 2017 to June 2017. Fifty-seven acute stroke patients with dysphagia (26 males and 31 females) were participating in a conventional swallowing therapy (50 minutes a day for 3 days per week). A functional oral intake scale (FOIS) and swallow function scoring system (SFSS) were used to determine an effectiveness of the swallowing therapy. FOIS and SFSS scores before the first therapy session and after the last therapy session were compared using a paired *t*-test.

**Results:**

The mean age of the patient was 69.5 ± 15.35 years. The period from stroke onset to the first swallowing therapy session was 7.5 ± 6.69 days. The number of therapy was 5.6 ± 2.83 sessions. Participants showed a significant improvement of the FOIS (mean score increased from 1.74 to 3.30 points, *P* = 0.001) and SFSS (mean score increased from 2.51 to 3.68 points, *P* = 0.001). Forty-two percent of patients with tube dependent change to total oral intake.

**Conclusion:**

Conventional swallowing therapy is an effective treatment in acute stroke with dysphagia.

## 1. Introduction

Dysphagia is a common problem in stroke patients. Dysphagia found up to 65% in acute stroke [1–3]. Acute stroke with dysphagia increases risk of aspiration pneumonia, prolonged length of hospital stay, increased health care costs, and mortality rate [4–8]. Furthermore, it may affect an emotion and a psychological health of patients.

Swallowing therapy has an important role in the recovery of dysphagia, prevention of aspiration, and improvement of quality of life [9, 10]. Conventional swallowing therapy consists of swallowing exercise and maneuver, postural and compensation technique, food and environmental modification, and alternative feeding [11]. Examples of swallowing exercise and maneuver are vocal cord exercise, effortful swallow, Shaker exercise, etc. Postural and compensation techniques such as chin tuck and head rotation. The easiest and safest food has a single consistency with high cohesiveness low adhesiveness and low hardness. Small bolus ingestion is easier than large bolus. Therefore, food consistency and bolus volume are modified and stepped up according to dysphagia severity. However, some patients need an alternative feeding such as nasogastric tube for prevention of aspiration and reducing dehydration and malnutrition. Nonconventional swallowing therapy that included neuromuscular electrical stimulation, EMG biofeedback, and neuromodulation is a method that requires special devices and not routinely used in our department.

Our rehabilitation medicine service routinely used a conventional swallowing therapy in acute stroke patients with dysphagia. Previous study in acute stroke with dysphagia showed an improvement of functional outcome, Functional Oral Intake Scale (FOIS), after 10 sessions of conventional swallowing therapy [12]. However, a psychometric assessment such as Functional Oral Intake Scale and Swallow Function Scoring System (SFSS) [13] was used for functional outcome measurement in the past 5 years. Therefore, the aim of this study is to determine an effectiveness of swallowing therapy in acute stroke patients with dysphagia admitted in our hospital and compare the outcome with other studies.

## 2. Methods

We retrospectively reviewed medical records of acute stroke patients with dysphagia in acute stroke unit whom participated a conventional swallowing therapy from January 2017 to June 2017. Dysphagia rehabilitation is a multidisciplinary approach. Swallowing team includes specialists from different disciplines, such as physiatrist, neurologist, occupational therapist, ward nurse, and nutritionist. Stroke patient in the acute stroke unit who has a suspected history presenting with swallowing dysfunction or a positive result of the modified water swallowing test was inserted a nasogastric tube and consulted to rehabilitation medicine service. The modified water swallowing test is used to detect aspiration. [14]. The patient is instructed to swallow a 3 ml water from a syringe. If the patient is unable to swallow, changes in breathing, cough, or wet-hoarse voice, they have a risk of aspiration. Physiatrist performs an evaluation and designs a goal-oriented rehabilitation program within 24 hours after the consultation. Physiatrist identifies a patient who has a proper medical condition prior swallowing therapy, suggests choices of swallowing exercise, and maneuvers to the occupational therapist. Then, stroke patient with dysphagia who has not contraindications for swallowing therapy is comprehensively evaluated by an occupational therapist include oromotor control, laryngeal function and elevation, gag reflex, dysarthria, and oral apraxia. The water swallowing test is used to detect aspiration [15]. A 3 ml water is placed under the tongue with a syringe. The patient is instructed to swallow. The test is positive if the patient is unable to swallow or experience cough, wet-horse voice, or dyspnea. If the patient is able to swallow without any signs, water volume is stepped up to 5 ml, 10 ml, and 20 ml. After that, physiatrist and occupational therapist will discuss and design an impairment-oriented swallowing therapy program for individual patients. After discharge from the acute stroke unit, some patients who have only swallowing dysfunction will participate an outpatient swallowing therapy. A stroke patient who has a disability other than dysphagia, such as self-care, ambulation will admit to a rehabilitation center.

An occupational therapist measures functional outcome, FOIS and SFSS, at the 1^st^ consultation and before discharge from the acute stroke unit. Instrumental swallowing assessment is not routinely performed in acute phase. In our hospital, physiatrist and occupational therapist perform the videofluoroscopic swallowing study in subacute or chronic stroke patients who have not responded to swallowing therapy.

This study protocol was approved by the ethics committee of Faculty of Medicine, Chulalongkorn University (IRB No. 635/60).

### 2.1. Conventional Swallowing Therapy

Conventional swallowing therapy is immediately conducted in a stable stroke patient with dysphagia. A conventional swallowing therapy does not require EMG biofeedback or neuromodulation devices. Physiatrist and occupational therapist will discuss and design a tailored-made conventional swallowing therapy that an impairment-oriented program for each patient. Patient participated in 50-minute conventional swallowing therapy once a day, 3 days per week during weekday.

Bed was adjusted to 60° or higher recline position before starting the therapy [16]. A conventional therapy is comprised of various techniques as follows: oromotor sensory stimulation [17, 18], the Mendelsohn maneuver [19], supraglottic swallow [20], range of motion exercise and oromotor strengthening [21], chin down/chin tuck [22], head turn to the weak side and tilt to the sound side [23], effortful swallow [24], and cough training [25]. Therefore, physiatrist and occupational therapist choose a different exercise, maneuver, or food modification based on an impairment of each patient.

Food modification with Xanthan gum thickeners (ThickenUp® Clear, Nestle Health Science) was used to modify the viscosity of food and liquid. We step up food modification from small to large bolus volume and from thick homogeneous food to thin liquid and mixed food. The patient uses tongue to remove the remaining food and throat clearing immediately right after feeding. NG tube was removed if the patient eats a single consistent oral diet [26].

### 2.2. Functional Outcome Measurement

Our hospital used the Functional Oral Intake Scale (FOIS) [12] and Swallow Function Scoring System (SFSS) [13] for functional outcome measurement. The FOIS is a statistically validated scale for assessing the oral intake of food and liquid in stroke patients. It is widely used to evaluate the functional oral intake of stroke patients with dysphagia. It categorized swallowing outcome into 7 levels (score 1–7). Level 1 to 3 is tube dependent. Level 4 to 7 is total oral intake. It is simple and convenient for therapist.

The SFSS is an assessment tool that measures the ability of liquid intake. It identified the consistency of liquid that a patient can swallow without aspiration. It categorized into 7 levels (score 0–6; from saliva aspiration to all liquid toleration). Although this scoring system is not validated, it may use as a tool for a food texture/consistency step up.

### 2.3. Statistical Analyses

Descriptive statistics presented as mean and standard deviation. Paired *t*-test was used to compare FOIS and SFSS scores before and after swallowing therapy. Pearson's correlation coefficient was used to evaluate the correlation between FOIS and SFSS scores. This study has statistically significant at 0.05 (confidence interval was taken as *P* < 0.05). Data analysis was performed by using SPSS (version 20).

## 3. Results

Demographic data of stroke patient is presented in Table 1. Fifty-seven acute stroke patients were included in this study (31 females, 26 males). Mean age at onset is 69.54 ± 15.35 years. Most of them are supratentorial stroke. Ninety-five percent had a positive result of 5 ml water swallowing test. All patients were nasogastric tube feeding before therapy. Mean time before starting the swallowing therapy is 7.48 ± 6.70 days. Each patient participated 5.6 ± 2.83 sessions.

**Figure 1:**
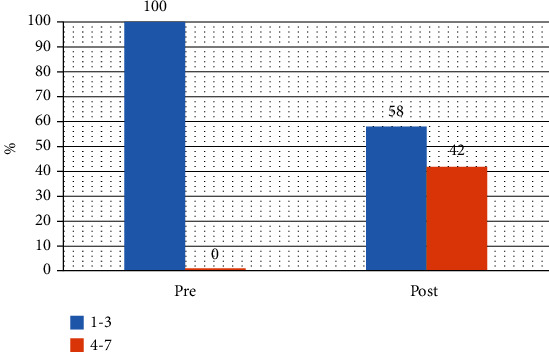
Functional Oral Intake Score (1–3: tube dependent, 4–7: total oral intake).

The mean score of FOIS and SFSS after swallowing therapy increases 1.56 points (*P* value = 0.0001) and 1.17 points (*P* value = 0.0001), respectively (Table 2). After therapy, 42% of patients with tube dependent change to total oral intake as shown in Figure 1. No aspiration pneumonia is found in our study. The correlation coefficient between FOIS and SFSS scores is 0.733.

## 4. Discussion

An effectiveness of conventional swallowing therapy in acute stroke with dysphagia is proved. The FOIS and SFSS scores increase at least 1 point before discharge. This study used daily swallowing therapy for 50 min, 3 times per week with an average of 5.6 sessions. FOIS score increases 1.56 points. The previous study used daily swallowing therapy for 60 min, 3 times per week for a total of 10 sessions. FOIS score increases 3.0 points [12]. The FOIS score of this study improves 0.28 points per session as same as 0.3 points per session in the previous study. The SFSS score of this study increases 1.17 points. SSFS score of a previous study increases 1.0 point [13]. That study used only thermal-tactile stimulation 5 times per week for 4 weeks. The SFSS score of this study improves 0.21 points per session. SSFS score of a previous study increases 0.05 points per session. However, we could not compare the improved SSFS score between the studies because different of stroke onset and therapy. We also found that the FOIS score is highly correlated with SFSS score at admission and discharge.

Of the patients admitted at the FOIS score 1–3 (NG tube dependent), 42% improved to the FOIS score 4–6 (no NG tube dependent) before discharge. 55% of acute (60% of cases) and subacute (40% of cases) stroke could off the NG tube after participating in a conventional swallowing therapy. Total time of swallowing therapy in that study is 2–11 hours [27]. In a previous study that used an early swallowing therapy in acute stroke, 64% of patients who requiring NG tube were discharged with no NG tube [28]. When comparing with the previous study, this study has less number of patients who could off the NG tube before discharge. However, the average time before starting the first swallowing therapy in this study is 7 days from an onset. There is not truly compare with the study in acute stroke who participated in early swallowing therapy, because 47% of acute stroke with mild dysphagia were improved over the first 7 days after onset [29]. After the first 7 days, 95% of consulted patients in this study have a positive water test and requiring NG tube feeding. Therefore, they were a stroke with moderate to severe dysphagia [30]. A patient who has an occasional aspiration, water/food aspiration, and saliva aspiration is classified as moderate to severe dysphagia.

There is no aspiration pneumonia during admission in this study. Summary of Cochrane review states that swallowing therapy in acute and subacute stroke with dysphagia may improve swallowing ability, reduce the proportion of patients with dysphagia, and reduce the incidence of pneumonia [31]. This study reports a similar result with that review [31].

Occupational therapist and physiatrist discussed and designed an individual program and trained them one-by-one. Multiple techniques were used according to patient's impairment. A mechanism of an example of therapeutic technique is clarified. Oral sensory stimulation with the sour and cold stimulus provides a sensory stimulation in the oral cavity and pharynx that reached the brainstem. The oral transit time is increased with this technique [32–34]. Posture, compensation techniques, swallowing exercise, and maneuvers strengthen oromotor muscles, shorten the food passage, and improve coordination of swallowing [19–25]. Modified liquid with Xanthan Gum thickener was adjusted to the proper level of swallowing in each patient. Consistency and bolus volume were stepped up as appropriate to reduce aspiration [35]. Therefore, the patients could participate in frequent swallowing training to prevent disuse of oropharyngeal muscles [36].

Aspiration pneumonia has not been found, and 42% of patients could off the NG tube in this study. A conventional swallowing therapy has an efficacy in improving the swallowing function and reducing aspiration risk. However, 58% have been used NG tube at discharge. There may be from the severity of dysphagia and suboptimal therapy session in some cases. Few patients have an apraxia that hinders training and need more repeatedly teaching. Fatigue is found in that case.

Limitations of this study are retrospective study, no instrumental dysphagia assessment information, and no long-term follow-up after discharge.

## 5. Conclusion

Conventional swallowing therapy is an effective treatment. Swallowing ability, proportion of the tube free, and the incidence of pneumonia improve in acute stroke patients with dysphagia.

## Figures and Tables

**Table 1:** Demographic and clinical characteristics of the patients.

Characteristics	Overall (*N* = 57)
Age, years, mean ± SD	69.54 ± 15.35
Gender, *n* (%)	
Male	26 (45.61)
Female	31(54.39)
Type of stroke, *n* (%)	
Hemorrhage	13 (22.80)
Ischemic	44 (77.20)
Stroke lesion, *n* (%)	
Right hemisphere	31 (54.39)
Left hemisphere	22 (38.60)
Brainstem	4 (7.01)
5 ml water swallowing test, *n* (%)	
Positive	54 (94.74)
Negative	3 (5.26)
Abnormal volitional cough, *n* (%)	36 (63.16)
Impaired tongue movement, *n* (%)	50 (87.72)
Reduced laryngeal elevation, *n* (%)	52 (91.23)
Dysarthria, *n* (%)	21 (36.84)
Hypo gag reflex, *n* (%)	9 (15.79)
Drooling, *n* (%)	3 (5.26)
NG tube insertion, *n* (%)	57 (100)
Swallowing therapy session, mean ± SD	5.58 ± 2.83

**Table 2:** Comparison of functional outcome before and after swallowing therapy.

Functional outcome	Mean ± SD	Mean different (95% CI)	*P* value
Functional Oral Intake Scale			
Before	1.74 ± 0.96	1.56 (-1.95, -1.17)	0.0001
After	3.30 ± 1.85		
Swallow Function Score System			
Before	2.51 ± 1.65	1.17 (-1.45, -0.9)	0.0001
After	3.68 ± 1.94		

## Data Availability

Data available on request through e-mail of corresponding author.
